# How Can We Better Prevent Obesity in Children?

**DOI:** 10.1007/s13679-015-0167-6

**Published:** 2015-07-16

**Authors:** Tommy L. S. Visscher, Stef P. J. Kremers

**Affiliations:** Research Centre for the Prevention of Overweight, Zwolle Windesheim University of Applied Sciences and VU University Amsterdam, PO Box 10090, 8000GB Zwolle, The Netherlands; Department of Health Promotion, NUTRIM School for Nutrition and Translational Research in Metabolism, Maastricht University, PO Box 616, 6200 MD Maastricht, The Netherlands

**Keywords:** Applied research, Community-based interventions, Evaluation research, Intervention Mapping, Process evaluation, Qualitative research

## Abstract

The aim of this review is to discuss the state of the art regarding the field of health promotion in the context of childhood obesity prevention in order to learn how we can better prevent childhood obesity. Challenges have been identified that exist within the different steps of health promotion programme development and implementation. Important steps forward include studying behaviours and determinants of behaviours as clusters, upgrading the importance of distal environmental factors in modelling determinants and understanding determinants as a dynamic system: a complex of interacting elements. An important note is that the process of implementation and the analysis thereof should more often come before the analysis of behaviours and the determinants of behaviour. In applied research, the expertise from the ‘real world’ practitioners should be used in an early stage to find out whether the answers on research questions really help us in preventing childhood obesity.

## Introduction

Readers of this special issue of *Current Obesity Reports* may well perceive the field of obesity prevention as long-standing and highly established. Indeed, we have made enormous progress in our understanding of obesity prevention during the last few decades. Conclusions regarding effectiveness of childhood obesity prevention differ across different review studies adopting different methodologies [1–3]. Although effective studies are available, a large number of interventions are still not proven effective. [1–3, 4•, 5]. Reviews showing an effect of childhood prevention do have difficulties explaining why and how childhood prevention is most effective.

Putting the prevention area in a historic perspective, it becomes clear that it is not surprising that we still need to learn how and why childhood prevention programmes are effective. Whereas William Harvey studied blood circulation 400 years ago [6], health education was introduced less than 100 years ago [7•] and preventive medicine targeting unhealthy behaviours and their determinants became important during the last 40–50 years [7•], after the Canadian Minister of Health Dr. Marc Lalonde and Sir Geoffrey Rose published their reports and visions on the importance of prevention and health promotion [8, 9]. It is no surprise that health behaviour became important during the last few decades only. Hence, not many more than 100 years ago, we did not live long enough to develop chronic diseases and energy supply and opportunities to be inactive became abundant during the last few decades. Why is it important to understand that the field of obesity prevention is only very young? Because reviewing the literature may reveal that our knowledge on important aspects of obesity prevention is still insufficient, and we have to prevent that such observations lead to pessimism or passive behaviour among interventionists. The message that our knowledge on childhood prevention needs to be further developed is a different message than a message that childhood prevention would not be effective.

As trends in childhood obesity are still rising across the globe [10•], and the impact of childhood obesity is tremendous during childhood and during later life [11], the aim of this review is to discuss the state of the art regarding the field of health promotion in the context of childhood obesity prevention in order to learn how we can better prevent childhood obesity.

## What Do We Need to Know?

According to planned health promotion strategies including the Intervention Mapping protocol [12•], prevention programme engineers need knowledge on the health issue we aim to target, and on the determinants, stakeholders and opportunities in society, before planning the implementation (Table 1). This reviews aims at describing what we know and what we do not know regarding these important issues in planning obesity prevention in children.

**Table 1:** How can we better prevent childhood obesity according to the different steps in the Intervention Mapping (IM) protocol [12•]?

IM step [12•]	The evidence	How can we better prevent childhood obesity?
Needs assessment	Childhood obesity is increasing. There are suggestions for downward trends.	Study long-term trends, severe categories and other measures than BMI.
Analysis of the health problem	Childhood obesity is associated with impaired health.	Study health consequences in the non-obese as well, do not focus on BMI alone and include non-medical consequences including stigmatisation.
Analysis of health-related behaviours	Multiple behaviours play a role in determining energy balance, and they differ across target groups.	Include patterns or clusters of behaviours in the analysis of behaviour.
Analysis of determinants of behaviours	Both personal and environmental determinants play a role.	Study determinants of patterns or clusters of behaviours, and study the interaction between personal and environmental determinants in quantitative studies.
Analysis of programme elements	Many components play a role. Studying components often comes after showing the effectiveness.	Studying programme components is important, even after having started, in order to further improve the programme when sustained, and for others across the globe.
Analysis of opportunities for implementation and sustainability	Knowledge and expertise regarding opportunities come from different fields of expertise. Opportunities are important determinants for the long-term success of prevention targeting a large numbers of professionals and individuals.	Analysis of opportunities is of key importance for successful implementation. Involvement of the ‘real world’ practitioners in defining the research questions is a promising way forward.
Analysis of process and effectiveness	Effectiveness studies are more common than process evaluations. One explanation is that process evaluation often relies on qualitative research methodologies. The RCT is often deemed as the most important evaluation design.	Convince reviewers and editors regarding the importance of qualitative studies. Include quantitative analyses in the process evaluation. Learn more regarding alternatives to the perfect randomised controlled trial. Improve monitoring systems.

## Analysis of Childhood Obesity Rates: The Needs Assessment

Most papers on childhood obesity prevention start reporting that prevention of childhood obesity is important because childhood obesity rates are increasing. Recent suggestions that childhood obesity rates may be levelling off or perhaps even decreasing thus imply a potential threat for future intentions to improve and sustain childhood obesity prevention programmes. Whether childhood obesity rates indeed show a levelling off or decrease has been a topic of several debates and reviews [10•]. Although levelling offs have been reported from methodologically strong studies, it is important to note that all but two studies [13, 14] showing decreases in childhood obesity were shorter than 5 years [10•]. Studies including severe classifications for childhood obesity and studies including the waist circumference did show increases, even if the same study showed a levelling off for the mean BMI or obesity defined by BMI [10•]. We can better prevent childhood obesity if we study the increase in childhood obesity in long-term studies, and when including the evaluation of severe categories and levels of skinfold thickness or the waist circumference.

## Studying Childhood Obesity as a Disease: Analysis of the Health Problem

BMI is the only measure for childhood obesity being used worldwide. This is a very good reason to keep on using BMI. Using BMI and agreed cut-off points is useful for comparisons across populations and comparisons within population over time [15]. The value of BMI as an indicator of increased risk, however, is likely to be exaggerated. Although BMI in childhood is a predictor of obesity in adulthood, skinfold thickness in childhood, for example, has been shown to be a better predictor of obesity in adulthood [16]. Doak et al. showed in their extensive review on school-based prevention programmes that studying skinfold thickness in evaluation studies is more likely to provide evidence for effectiveness of those interventions than studying the BMI alone [2]. Thus, childhood prevention programmes focussing on BMI alone are likely to underestimate the real effect of childhood obesity prevention.

Childhood obesity is now well understood as a disease [11]. Obese children have been described to be at increased risk of health complications including impaired glucose tolerance, elevated blood pressure and impaired glucose levels [11, 17–19]. As childhood obesity is still relatively rare, although increasing, in most countries, it may well be anticipated that childhood obesity complications are also rare. Here, it is important to understand the lessons regarding the continuum of disease risk that we learned from Rose [9] and Pickering [20]. Their examples showed that every increase in blood pressure was associated with a further increased risk of complications, also when blood pressure was below the agreed definition for hypertension. The same is true for the degree of overweight. For every increase in BMI, children show higher levels of known risk indicators for developing diabetes and cardiovascular diseases [17–19].

Moreover, childhood obesity needs to be understood as a complication that exceeds the medical domain. Obese children are more often subject of bullying, and their social life is often impaired, by which they more often experience an impaired quality of life, than healthy weight children. Stigmatisation plays an important role [21••]. Thus, as prevention of childhood obesity starts with studying the problem and related complications that we want to prevent, we can better prevent childhood obesity if we know better what our aims are when preventing childhood obesity and when we accompany our efforts with other measures than BMI alone.

## Analysis of Health-Related Behaviour

Studying determinants of childhood obesity is complicated. Hence, whereas we know that we have to look into energy balance-related behaviours, other mechanisms play a role too, including short sleep duration [22]. At the same time, understanding that so many behaviours play a role in causing childhood obesity, it is clear that any single behaviour plays a little role only. The food industry is very keen on explaining that the sales of their ‘single snack’ or ‘single soft drink’ is only one player amongst so many others and should well fit ‘within a healthy pattern of energy intake’. But what is a single behaviour, and should we focus on single behaviours? They answer is most likely ‘no’. Studies show that energy balance-related behaviours cluster [23]. A change in a single behaviour may induce changes in another, clustered, behaviour. And we know that the industry behind the single snacks and the single soft drinks, as well as the industry of inactivity, are massive and could well be considered as an important cluster. We can better prevent childhood obesity if we study behaviours as clusters of behaviours and look at behavioural patterns rather than at isolated acts.

## Analysis of Determinants of Behaviour

Whereas we understand that the behaviours leading to childhood obesity are numerous, it is important to understand that every single behaviour is determined by its own determinants of behaviour that are thus numerous, too. From the other hand, in line with the reasoning in terms of clusters as indicated above, the determinants of behaviour may be easier to study and understand when they are studied as determinants of behavioural clusters. It seems fair to hypothesise that if behaviours cluster, determinants of those behaviours cluster, too [24, 25, 30•].

Although research in the area of determinants of obesity is continuing at a rapid pace, true advancements in this research field are lacking. One of the reasons lies in the dominance of socio-cognitive behavioural models in health behaviour theory and the tendency of researchers to stick to one-dimensional, linear and isolated research frameworks. Moreover, the study of health behaviour in isolation from the broader environmental context is incomplete. Behavioural determinant studies are executed in our usual way of doing things: ‘same old, same old’ [26], applying a rational linear socio-cognitive approach. To advance the obesity prevention field, we need to deviate from the traditional socio-cognitive approach in two important respects. First, we need to downgrade the importance of linear socio-cognitive processes in determining energy balance-related behaviours. Second, we need to upgrade the importance of distal environmental factors in modelling determinants [24, 27–29, 30•, 31, 32•, 33, 34•]. Distal environments function as driving factors in the causal chain, but also as higher order moderators of more proximal environmental determinants [30•, 35•].

The present-day living environment has often been labelled ‘obesogenic’, but the environmental perspective has not yet received the same degree of theoretical attention in the study of determinants as the individual perspective. In the ecological perspective of health behaviour [36], context relates to multiple spheres of the social and physical influences (micro-, meso-, exo- and macro-levels). Ecological studies in the field of child development have shown that the impact of micro-level factors (e.g. parental support for a child to play outside) on individual behavioural developmental variability can vary as a function of contextual macro-level conditions (e.g. the presence of playgrounds in the neighbourhood) [2, 37]. The operation of such higher order moderation processes underlines the importance of distal, so-called upstream determinants of behaviour. The existence of higher order moderation has been suggested in the field of physical activity and dietary behaviour [38, 39], but, to date, distal factors have typically been operationalised as confounders in causal chain determinants research.

The contemporary mechanistic orientation results from the prevailing implicit assumption that complex phenomena are reducible to their basic elements, and that once we understand the basic elements, we can understand everything else. However, this assumption does not sufficiently reflect the complexity of the impact of the obesogenic environment. An accurate reflection would require the view of context as a dynamic system. The concept of system refers to a ‘complex of interacting elements’ [40] or a ‘group of parts that are interacting according to some kind of process’ [41]. What is common to the various definitions of a system is not the characteristics of the individual units or parts but rather the extent and nature of linkages or interrelationships among the various units [42]. The impact of a system is more than just the sum of the individual parts. The operation of any one element in a system depends on the existence and operation of other elements in the system. This implies that the impact of the fast-food restaurant around the corner of one’s street cannot be understood by mechanistically modelling it by correcting for all other potential determinants in the causal chain (e.g. neighbourhood characteristics, personal attributes, demographics), but by examining the system conditions under which the restaurant has an impact.

It is clear that single aspects in the environment cannot be blamed in isolation as the determinant of obesity. When determinants are clustered, as for example is being done intentionally by the marketing industry, they could have a huge impact on children’s behaviour. Millions are spent to determine people’s food choices, including those of children. Cairns et al. conclude from their summary of earlier reviews that food promotions have a direct effect on children’s nutrition knowledge, preferences, purchase behaviour, consumption patterns and diet-related health [43•]. The effect of food promotion can have an effect on children’s, purchase behaviour and consumption independently of other influences [44, 45]. Current marketing practice predominantly promotes low nutrition foods and beverages [43•]. Unfortunately, little progress has been made towards rebalancing the food marketing landscape [43•]. We can prevent better childhood obesity if we study determinants of clustered behaviours and if the marketing of unhealthy foods towards children is prevented.

## Analysis of Programme Components

Whereas the planning of health promotion often includes the study of programme components, it is important to understand that childhood obesity prevention programmes are often ‘Black boxes’ in which creativity plays a role and in which the needs of populations and subpopulation often ask for ad hoc approaches. At least, the majority of community-based prevention programmes have in common that they exist of many and different components. In line with our argumentation regarding the importance of studying systems, Rutter replied to the question ‘What is the single most important intervention to reduce childhood obesity?’ that the most important intervention is to understand that there is no single most important intervention [46]. Therefore, the analysis of programme components should not be performed in isolation. Also, potentially important components should not be studied in direct relation with the final outcome alone.

The analysis of programme components has proven to be hard. Whereas evidence for effectiveness of EPODE, for example, was spreading rapidly across the globe [47], the understanding of important components of EPODE came later [48]. The analysis of effectiveness of EPODE was quite straightforward. Changes in the prevalence of childhood obesity in two EPODE cities were compared with changes in obesity rates on control towns [47]. Although it took some time and efforts to publish the results of EPODE, because reviewers were asking for a randomised controlled trial design, the dissemination of the results after publication went well. Opening the ‘Black box’ of the EPODE programme in order to learn the dynamics and key aspects of EPODE took considerably more time [48]. A very important lesson from the EPODE programme thus was that it is not crucially important to know the specific elements of the prevention programme exactly before being effective. Knowledge regarding the key elements and dynamics is now used for future implementation of EPODE programs on a wide scale.

Note that we do not advocate to refrain from studies that are specifically aiming to identify single, isolated determinants of behaviour. Such studies can inform our knowledge of potentially important elements in the obesity system, and they can inform the contents of systems-based interventions to prevent obesity. Nice examples of such an isolated approach include an elegant randomised controlled trial showing that replacing sugars by non-energetic sweeteners in soft drinks had a beneficial effect on children’s body mass index [49]. A randomised controlled trial on reducing television viewing and computer use showed beneficial effects on BMI in young children [50].

We can better prevent childhood obesity if we combine carrying out studies towards essential intervention components that have been proven effective in randomised controlled trials and if we improve our understanding of the importance of the contextual and interactive nature of behavioural determinants in the field of childhood obesity. In addition, we need to acknowledge the informational value of alternatives to the perfect randomised controlled trial design when evaluating community-based programmes.

## Analysis of Opportunities for Implementation and Sustainability

It is a pity, or perhaps a severe barrier for effective childhood obesity prevention, that the analysis of opportunity often comes after the analysis of behaviour and the analysis of determinants of behaviour. The Intervention Mapping protocol [12•] is often misinterpreted as if an optimal approach requires a consecutive order of these analyses. Instead, optimal approaches often require a dynamic process of studying the needs, studying behaviours and determinants of behaviours, and studying opportunities.

Studies regarding the opportunities often lead to conclusions that the community-based approach is the best option. Understanding that so many behaviours and so many determinants play a role, including environmental determinants, it is well understood that enabling community capacity and empowerment of individuals should be key factors in childhood obesity prevention.

As we know, showing evidence for effective childhood obesity prevention is well possible in relatively small study settings in which professionals and participants know what is expected and in which both professionals and individuals are determined to be successful. But the real challenge is to have a large impact in the community, meaning, to reach as many communities, professionals and individuals as possible [51].

The real drivers to tackle childhood obesity are to be found within a wide variety of environmental determinants [27–29], being influenced by a wide variety of stakeholders. A multidisciplinary, multisectoral or integrated approach is essential in community-based prevention of childhood obesity. Expertise from many and various stakeholders is needed for successful implementation of childhood obesity prevention programmes [32•, 52]. These stakeholders do not necessarily have to consider the health of the child as their main interest [53•]. Summerbell et al. concluded from their study of reviews that childhood obesity prevention programmes should not focus on the educational setting alone. They clearly confirmed the importance of the role of local governments, non-governmental organisations and the media [4•]. Understanding that the multidisciplinary approach is important, in which expertise comes together from different angles, it is clear that all relevant knowledge and expertise is unlikely to be available within one stakeholder. Thus, childhood obesity prevention could really improve if we talk with and learn from the stakeholders first. Further, it has been argued before that findings can be highly relevant for those in the academic world, but are sometimes less relevant/feasible for ‘real world’ practitioners [54•]. We can better prevent obesity in children when studying aspects of implementation and studying behaviour and determinants of behaviour more dynamically, and when we define research questions regarding implementation issues together with the ‘real world’ practitioners, which is a basic principle in applied sciences.

## Analysis of the Process and Effectiveness Evaluation

An appropriate process evaluation, studying the ‘Black box’ revealing the effective strategies and opportunities, is important to let prevention programmes become successful elsewhere and to further improve sustained implementation over the years.

Process evaluation is often associated with qualitative studies. These qualitative studies teach us lessons from the experts in the field: the communities, individuals in the communities and the professionals working in those communities. These studies are hard to publish, but are of enormous importance in getting further with childhood obesity prevention [55]. At the same time, with the enormous increase in community-based childhood obesity prevention programmes worldwide, quantitative analyses may also be used as part of the process evaluation. Hence, if process evaluation means the learning about processes and elements within childhood prevention programmes, the increase in childhood obesity programmes is likely to imply that the number of comparable processes and elements is also increasing, enabling quantitative analyses.

A major issue in studying the effectiveness is the fact that the perfect randomised controlled trial (RCT) is virtually impossible in childhood obesity prevention evaluations [56]. Attention to design issues will ultimately lead to more successful, cost-effective trials and more rapid movement toward efficacious and effective obesity prevention programmes [56]. Where controlling is a major issue in medical drug trials for instance, researchers aim at controlling the intervention arms such that arms are completely the same, except for the intake of the drug. In community-based prevention, however, it is the creativity enabling opportunities that is an important feature that should not be controlled. Here, we have a challenge to improve ourselves convincing reviewers and editors the value of other evaluation designs than the RCT [55]. Alternatives could, for instance, include action-oriented research. Action-oriented research is defined as ‘the study of a social situation with a view to improving the quality of action within it’ [57]. The methodology enables researchers and their participants to learn from each other through a cycle of planning, action, observation and reflection. Observations in research may thus lead to changes in the implementation or intervention refinements. Data gathering typically involves a mixed-method approach, combining quantitative (e.g. questionnaires and checklists) with qualitative research techniques (e.g. interviews, observations).

Whereas studying both facilitating as inhibiting factors is common in qualitative process evaluation, reporting adverse effects in quantitative effectiveness evaluations is not common [4•] but should be advocated [2]. We can better prevent childhood prevention if efforts improve regarding monitoring and if we improve and disseminate our understanding of alternatives to the perfect randomised controlled trial.

## Conclusions

We conclude that our understanding of childhood obesity, energy balance-related behaviours, determinants of behaviour and effective components of community-based prevention programmes is young, perhaps immature. A strong involvement of the ‘real world’ practitioners in performing applied sciences is a promising way forward. We hope that consideration of issues discussed in this review may help to improve the efficiency and effectiveness of efforts that are aimed to influence (elements within) the system that impacts on childhood obesity.
